# Vision Status in Older Adults: The Brazilian Amazon Region Eye Survey

**DOI:** 10.1038/s41598-018-19338-6

**Published:** 2018-01-17

**Authors:** Solange R. Salomão, Adriana Berezovsky, João M. Furtado, Arthur G. Fernandes, Sergio Muñoz, Nívea N. Cavascan, Marcela C. Cypel, Cristina C. Cunha, Galton C. Vasconcelos, Márcia R. K. H. Mitsuhiro, Paula Y. Sacai, Mauro Campos, Paulo H. A. Morales, Marcos J. Cohen, Jacob M. Cohen, Sung E. S. Watanabe, Rubens Belfort

**Affiliations:** 1 Departamento de Oftalmologia e Ciências Visuais, Escola Paulista de Medicina, Universidade Federal de São Paulo – UNIFESP, São Paulo, SP Brazil; 2 Departamento de Oftalmologia, Otorrinolaringologia e Cirurgia de Cabeça e Pescoço, Faculdade de Medicina de Ribeirão Preto da Universidade de São Paulo, Ribeirão Preto, SP Brazil; 3 Departamento de Salud Publica, Universidad de La Frontera, Temuco, Chile; 4 Faculdade de Medicina da Universidade Federal do Pará – UFPA, Belém, PA Brazil; 5 Departamento de Oftalmologia e Otorrinolaringologia, Faculdade de Medicina da Universidade Federal de Minas Gerais – UFMG, Belo Horizonte, MG Brazil; 6 Divisão de Oftalmologia, Departamento de Cirurgia, Faculdade de Medicina da Universidade Federal do Amazonas – UFAM, Manaus, AM Brazil

## Abstract

Older adults living in remote areas with limited access to health services are at higher risk to develop visual impairment and blindness. We conducted a population-based survey to determine the vision status in subjects 45 years of age and older from urban and rural areas of Parintins city, Brazilian Amazon Region. Participants underwent ophthalmic examination, including uncorrected (UCVA), presenting (PVA) and best-corrected visual acuity (BCVA). Vision status was described as lines of visual acuity (VA) impairment and lines of VA improvement from UCVA to BCVA and from PVA to BCVA in the better-seeing eye. A total of 2384 subjects were enumerated, 2041 (85.6%) were examined, with reliable VA measurements obtained from 2025 participants. Vision status in lines of VA impairment was (mean ± standard deviation): 3.44 ± 3.53 for UCVA, 2.85 ± 3.52 for PVA and 1.50 ± 3.51 for BCVA. Female gender, older age and lower education were associated with ≥6 lines of UCVA impairment. Lines of improvement ≥3 was found in 626 (30.9%) participants and associated with female gender and rural residency. In conclusion, a third of participants could have at least three lines of VA improvement with proper refraction. Strategies to improve access to eye care and affordable glasses are needed.

## Introduction

The magnitude and burden of visual impairment (VI) and blindness usually are estimated through population-based epidemiological studies based on visual acuity cut-off criteria using either best-corrected or presenting distance visual acuity (VA)^1^. Most studies detail only the visual acuity levels of those blind or visually impaired, and little is known on the visual status of those considered with “normal” vision^2^. The distribution of visual acuity and impairment are of intrinsic value in defining the vision status of a population^3^.

Over the last decade there was a shift between the use of “best corrected” to a current use of “presenting” distance visual acuity (visual acuity measurement using the refractive correction in use, if any) for blindness^4,5^, but most studies do not report uncorrected VA. A more detailed analysis across the entire visual acuity range from 20/20 to no light perception could lead to a better understanding of the vision status of specific populations, and advocate for medical interventions in those at risk of developing visual impairment in the future.

The present data come from a recent population-based survey of visual impairment and blindness among adults in the city of Parintins located in the Brazilian Amazon Region (BARES)^6^. The city of Parintins with a 2010 census population of 102,718, is fairly representative in sociodemographic terms of the population of the Brazilian Amazon Region population. (The Brazilian Amazon Region has a total population of 24,700,000 with 72.2% living in urban areas.) Adults 45 years or older were 17.1% of the population in Parintins city (Census 2010) and 24% in the BARES sample with 44.7% of these male vs 44.9% throughout urban areas of the Brazilian Amazon Region. Literacy is 89.8% in Parintins, compared to 89.7% for the Brazilian Amazon Region. Our objective was to describe the vision status and improvement from uncorrected and presenting vision status to best-corrected vision status in urban and rural populations from the Brazilian Amazon.

## Methods

### Study Population

BARES is a population-based, cross-sectional epidemiological study designed to examine the prevalence and causes of vision impairment and blindness in a noninstitutionalized, population-based sample of older Brazilian Amazonians. Parintins is a 102,000 inhabitants city located at 02°37’42”S and 56°44′09″W, in the center of the Brazilian Amazon Region on the sides of the Amazon River, with hot and humid tropical climate. It can be reached by air (300 km) and about 420 km by boat from Manaus. The study population consisted of residents 45 years or older, living in 20 randomly selected clusters (14 urban and 6 rural) in Parintins city. The study population was selected through cluster sampling based on 2010 Census data^5^. In defining sampling frame clusters, census sectors with fewer than 70 persons 45 years of age or older were grouped with an adjacent sector and sectors with more than 140 persons 45 years of age or older were segmented, producing 154 clusters with 70 to 140 persons 45 years of age or older.

Sample size requirements were calculated on the basis of estimating an expected blindness prevalence of 6% (50% higher than that used in an earlier study performed in Southeastern Brazil, the São Paulo Eye Study - SPES) within an error bound (precision) of 20% with 95% confidence interval^7,12^–15. Assuming an examination response rate of 85%, allowing for an arbitrary 50% increase in sample size to accommodate possible inefficiencies associated with the cluster sampling design, and adjusting for the finite population factor, a sample size of 1393, with an increase to 2459 persons 45 years or older was required. Accordingly, 20 clusters (14 urban and 6 rural), consisting of 18 census sectors, were randomly selected with equal probability from the 154 sampling units. Further details on sampling plan, and baseline demographic data have been reported elsewhere^6^. In brief, a door-to-door enumeration was performed and eligible subjects were informed about the study and invited to participate in a clinical ophthalmic examination.

### Ethical approval and informed consent

The institutional review board/ethics committees both from Universidade Federal de São Paulo (UNIFESP) and from Universidade Federal do Amazonas (UFAM) approved the study protocol. The study was carried out in accordance to the tenets of the Declaration of Helsinki. Written informed consent was obtained from all participants after explanation of the nature and possible consequences of the study.

### Sociodemographic and clinical data

The enumeration form included household address, phone number, and a roster of those living in that home along with their gender, age and schooling. All eligible individuals (adults 45 years of age and older) were invited and scheduled for a detailed eye examination. Written informed consent was obtained at the examination site, followed by VA measurements for distance, refraction, and an eye examination. The examination protocol was similar to the one used in SPES^7^, including VA testing, which was performed in a standardized manner at the local eye clinic for urban participants and at an itinerant eye examination center for those living in rural clusters^6^. In-home eye exam was offered for those who could not come to the clinic and it was performed with portable equipment.

### Visual Acuity Testing

Ophthalmic technologists measured from each eye presenting distance visual acuity (PVA), with spectacles if the participant presented with them followed by uncorrected distance visual acuity (UCVA), using retro-illuminated logarithm of the minimum angle of resolution tumbling E charts at 4 m distance. Details about visual acuity measurement procedure can be found elsewhere^7^. During the enumeration process participants were asked about spectacle usage and advised to bring their glasses to the eye exam. Glasses were cleaned before visual acuity measurement when needed. Best-corrected visual acuity (BCVA) was determined for each eye after auto-refraction followed by subjective refraction performed by an ophthalmologist.

### Vision Status – Lines of Vision Impairment and Improvement in Lines of Vision Impairment

Vision status was defined for each eye by lines of vision impairment on the ETDRS chart from 0 to 16, with the number increasing as VA deteriorated. Accordingly, VAs of 20/20, 20/25, 20/32 and 20/40 were represented by line numbers 0, 1, 2 and 3, respectively. VA of 20/200 was represented by line number 10, with line 11 corresponding to VA of 20/250; line 12 corresponding to VA of 20/320; line 13 corresponding to VA of 20/400; line 14 to VA of 20/500; line 15 to VA of 20/640 and line 16 to VA ≤ 20/800.

Improvement in lines of vision impairment was calculated for each eye as the UCVA line number minus the line number at BCVA; thus, a positive line change indicated an improvement of vision between UCVA and BCVA and a negative change indicated worsening of vision between UCVA and BCVA.

Visual acuity measurements were categorized as: normal vision, ≥20/32; near-normal vision (mild visual impairment), <20/32 to ≥20/63; visual impairment, <20/63 to ≥20/200; moderate blindness, <20/200 to ≥20/400; and severe blindness, <20/400. Those with 20/20 vision in both eyes were noted.

Multiple logistic regression was used to investigate the association of categories of age (45–54, 55–64; 65–74 and ≥75 years), gender, categories of schooling (none, less than primary, primary, secondary, high school or higher) and urban/rural residence with participation in the study and with vision impairment. Statistical analyses were performed using Stata/SE Statistical Software, Release 14.0, 2015 (Stata Corp, College Station, Texas, USA)^8^. Confidence intervals (CI) for prevalence estimates and regression odds ratios (OR) were calculated taking cluster sampling design effects into account. P values ≤ 0.05 were considered statistically significant.

### Data Availability

The datasets generated during and/or analysed during the current study are available from the corresponding author on reasonable request.

## Results

During the period from March 2014 to May 2015, a total of 2041 participants were recruited and completed an eye examination. Of the 9931 residents, 2384 (24.0%) were eligible (aged 45 years and older) for BARES. Of these, 8 (0.3%) refused to participate, 335 (14.1%) did not show up for the clinical examination, and 2041 completed an ophthalmic examination, resulting in a participation rate of 85.6% (2041/2384). Demographic characteristics of the study population were described in detail previously^6^. Reliable visual acuity measurements in both eyes were obtained from 2025 participants. Table 1 shows the distribution of gender, age and schooling of the participants from rural and urban areas. Examination response was associated with female gender (odds ratio [OR], 1.44; 95% confidence interval [CI]:1.06–1.94). Age between 65–74 years (OR, 1.84; 95% CI: 1.29–2.62) and rural residency (OR, 1.91; 95% CI: 1.24–2.94) were associated with participation in the study. Higher education was associated with examination response for the categories “less than primary” (OR, 2.16; 95% CI: 1.15–4.04); “primary” (OR, 2.57; 95% CI: 1.55–4.24); “secondary” (OR, 2.53; 95% CI: 1.64–3.93) and “high school or higher” (OR, 2.91; 95% CI: 1.58–5.35).

**Table 1:** Study population by gender, age, and education in rural and urban sites in those with reliable VA measurements in one or both eyes.

	Gender		Age (years)				Education					All
	Male	Female	45–54	55–64	65–74	75+	None	<Primary	Primary	Secondary	≥High School	
**Urban**												
Enumerated	661 (46.9)	749 (53.1)	538 (38.2)	454 (32.2)	229 (16.2)	189 (13.4)	93 (6.6)	354 (25.1)	375 (26.6)	252 (17.9)	336 (23.8)	1410 (100.0)
Examined*	532 (45.5)	637 (54.5)	442 (37.8)	382 (32.7)	194 (16.6)	151 (12.9)	76 (6.5)	287 (24.5)	314 (26.9)	210 (18.0)	282 (24.1)	1169 (100.0)
% Examined	80.5%	85.0%	82.2%	84.1%	84.7%	79.9%	81.7%	81.1%	83.7%	83.3%	83.9%	83.5%
**Rural**												
Enumerated	537 (55.1)	437 (44.9)	400 (41.1)	294 (30.2)	169 (17.3)	111 (11.4)	209 (21.5)	277 (28.4)	287 (29.5)	93 (9.5)	108 (11.1)	974 (100.0)
Examined*	460 (53.7)	396 (46.3)	345 (40.3)	263 (30.7)	154 (18.0)	94 (11.0)	155 (18.1)	254 (29.7)	261 (30.5)	83 (9.7)	103 (12.0)	856 (100.0)
% Examined	85.7%	90.6%	86.3%	89.5%	91.1%	84.7%	74.2%	91.7%	90.9%	89.2%	95.4%	87.9%
**Total**												
Enumerated	1198 (50.2)	1186 (49.8)	938 (39.3)	748 (31.4)	398 (16.7)	300 (12.6)	302 (12.7)	631 (26.5)	662 (27.7)	345 (14.5)	444 (18.6)	2384 (100.0)
Examined*	992 (49.0)	1033 (51.0)	787 (38.9)	645 (31.8)	348 (17.2)	245 (12.1)	231 (11.4)	541 (26.7)	575 (28.4)	293 (14.5)	385 (19.0)	2025 (100.0)
% Examined	82.8%	87.1%	83.9%	86.2%	87.4%	81.7%	76.5%	85.7%	86.8%	84.9%	86.7%	84.9%

Data are given as number (percentage) of participants*. In 16 examined participants reliable visual acuity measurements could not be obtained in either eye: 11 urban (6 females and 5 males) and 5 rural (3 females and 2 males).

In the 2025 participants with reliable VA, 1293 (63.4%) presented without glasses; glasses for distance only were used by 23 (1.1%) participants; glasses for both near and distance were used by 480 (23.7%) participants; and glasses for near only were used by 229 (11.3%) participants.

**Figure 1:**
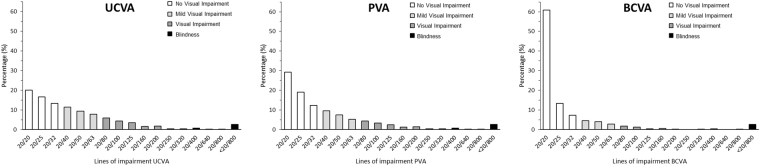
Lines of vision impairment in the better-seeing eye considering uncorrected (UCVA), presenting (PVA) and best-corrected (BCVA).

Overall, lines of VA impairment in the better-seeing eye (N = 2025) was (mean ± standard deviation): 3.44 ± 3.53 with UCVA, 2.85 ± 3.52 with PVA and 1.50 ± 3.51 with BCVA. The prevalence of 6 or more lines of uncorrected vision impairment was 21.6% (95% CI: 19.8 to 23.4); 17.0% (95% CI: 14.8 to 19.2) for presenting vision impairment and 7.3% (95% CI: 6.1 to 8.5) for best-corrected vision impairment. In multiple logistic regression modeling, 6 or more lines of vision impairment was associated with older age for uncorrected, presenting and best-corrected vision in the better-seeing eye (Table 2). Female gender was associated with 6 or more lines of only uncorrected vision impairment. Higher schooling categories (primary, ≥high school for uncorrected vision; primary, secondary, ≥high school for presenting vision; ≥high school for best-corrected vision) were associated with lower odds to have 6 or more lines of vision impairment. Rural residence was associated with 6 or more lines of vision impairment for both presenting and best-corrected vision. Figure 1 shows the distribution of lines of impairment in the better-seeing eye based on UCVA, PVA and BCVA along with vision status categories. In this cohort 20/20 vision in both eyes was found in 215 (10.6%) participants for UCVA, in 313 (15.5%) for PVA and in 893 (44.1%) for BCVA.

**Table 2:** Prevalence of best-seeing eye uncorrected, presenting and best-corrected vision impairment ≥6 lines by age, gender, schooling and residence.

	Number Examined	Prevalence UCVI	UCVI	Prevalence PVI	PVI	Prevalence BCVI	BCVI
		N (%)	OR [95% CI]	N (%)	OR [95% CI]	N (%)	OR [95% CI]
**Age (years)**							
45–54	787	75 (9.5%)	Reference	45 (5.7%)	Reference	9 (1.1%)	Reference
55–64	645	112 (17.4%)	1.67 [1.28; 2.17]^*^	71 (11.0%)	1.61 [1.14; 2.26]^†^	15 (2.3%)	2.62 [1.00; 1.91]^†^
65–74	348	108 (31.0%)	3.13 [2.19; 4.48]^*^	95 (27.3%)	3.81 [2.44; 5.97]^*^	38 (10.9%)	11.11 [5.80; 21.26]^*^
≥75	245	143 (58.4%)	9.27 [6.93, 12.39]^*^	134 (54.7%)	11.21 [7.37, 17.05] ^*^	86 (35.1%)	38.74 [16.43, 91.36]^*^
**Gender**							
Male	992	221 (22.3%)	Reference	190 (19.1%)	Reference	83 (8.4%)	Reference
Female	1033	217 (21.0%)	1.29 [1.05; 1.58] ^†^	155 (15.0%)	0.93 [0.74; 1.18]	65 (6.3%)	0.78 [0.55; 1.11]
**Schooling**							
None	231	96 (41.6%)	Reference	91 (39.4%)	Reference	50 (21.6%)	Reference
Less than Primary	541	160 (29.6%)	0.80 [0.59; 1.07]	141 (26.1%)	0.88 [0.63; 1.23]	59 (10.9%)	0.83 [0.44; 1.54]
Primary	575	95 (16.5%)	0.56 [0.41; 0.75]^*^	73 (12.7%)	0.52 [0.34; 0.79]^†^	29 (5.0%)	0.57 [0.24; 1.39]
Secondary	293	40 (13.6%)	0.66 [0.40; 1.10]	20 (6.8%)	0.47 [0.25; 0.90]^†^	6 (2.0%)	0.41 [0.08; 2.01]
≥High School	385	47 (12.2%)	0.47 [0.27; 0.82]^†^	20 (5.2%)	0.24 [0.14; 0.42]^*^	4 (1.0%)	0.23 [0.59; 0.88]^†^
**Residence**							
Urban	1169	226 (19.3%)	Reference	165 (14.1%)	Reference	80 (6.8%)	Reference
Rural	856	212 (24.8%)	1.27 [0.97; 1.66]	180 (21.0%)	1.53 [1.06; 2.21]^†^	68 (7.9%)	1.38 [1.00; 1.91]^†^
ALL	2025	438 (21.6%)		345 (17.0%)		148 (7.3%)	

UCVI – uncorrected vision impairment; PVI – presenting vision impairment; BCVI – best-corrected vision impairment; OR – adjusted odds ratio; CI – confidence interval.

^*,†^p < 0.001.

^†^p ≤ 0.05.

**Figure 2:**
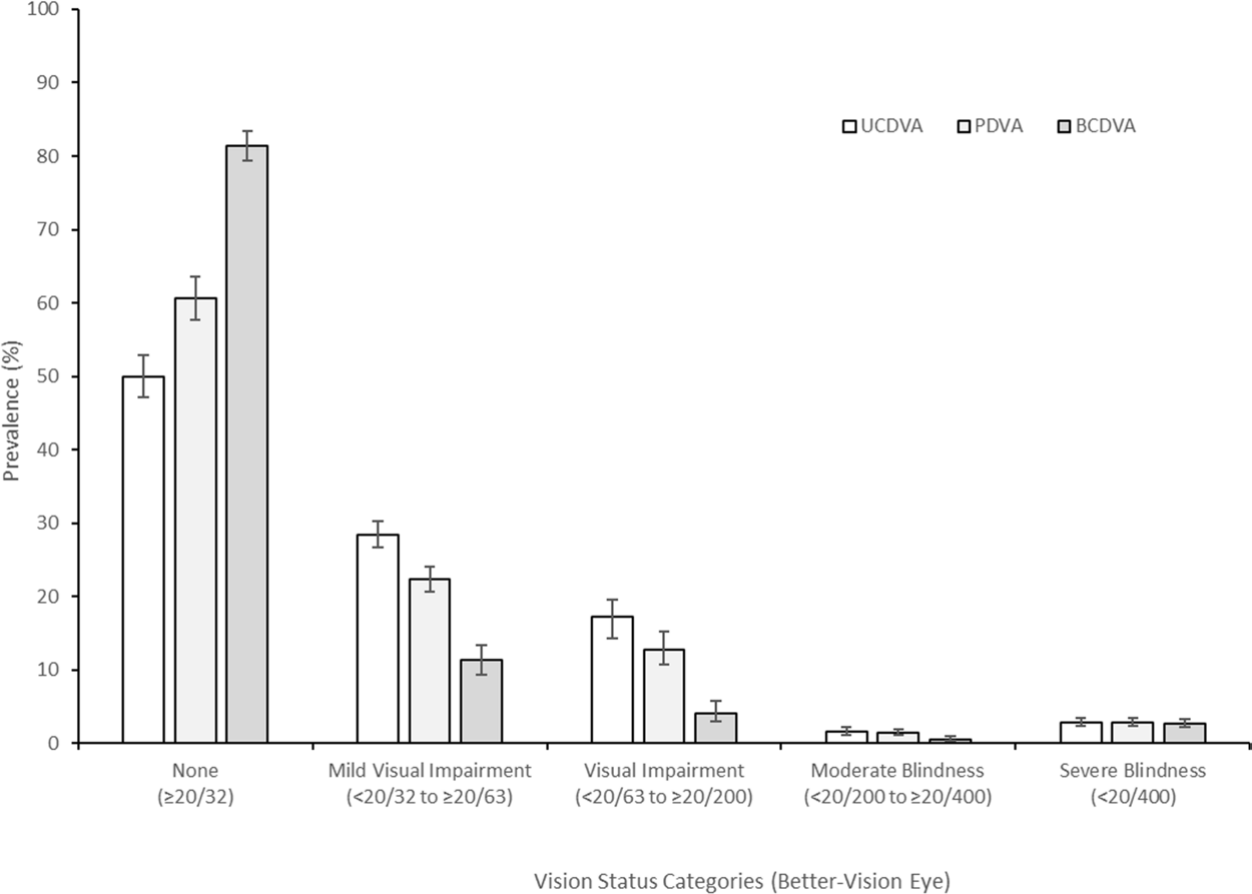
Prevalence of visual status categories for uncorrected (UCVA), presenting (PVA) and best-corrected (BCVA) in the better-seeing eye. Error bars represent 95% confidence intervals.

Uncorrected normal vision in the better-vision eye (VA of 20/32 or better) was found in 49.9% [95% Confidence Interval (CI): 47.1–52.8] of the sample increasing to 60.6% [95% CI: 57.7–63.5] for presenting VA and to 81.4% [95%CI: 79.3–83.4] for best-corrected VA. The prevalence of uncorrected mild visual impairment (20/40 to 20/63) in the better-seeing eye was 28.4% [95% CI: 26.7–30.3]; 22.3% [95% CI: 20.7–24.0) for presenting VA, and 11.3% [95% CI: 9.4–13.4] with best correction. The prevalence of uncorrected visual impairment (<20/63 to 20/200) was 17.2% [95% CI: 14.29–19.8]; 12.8% [95% CI: 10.8–15.2] for presenting VA and 4.1% [95% CI: 2.9–5.7) with best correction. The prevalence of uncorrected moderate blindness (<20/200 to 20/400) in the better-vision eye was 1.58% [95% CI: 1.15–2.17]; 1.38% [95% CI: 1.04–1.84] for presenting VA and 0.54% [95% CI: 0.31–0.94) with best correction. The prevalence of uncorrected severe blindness (worse than 20/400) in the better-seeing eye was 2.86% [95% CI: 2.34–3.50]; 2.81% [95% CI: 2.30–3.44] for presenting VA and 2.67% [95% CI: 2.18–3.25) with best correction. These results are presented in Fig. 2.

Table 3 shows the line-by-line distribution of UCVA in the better-seeing eye along with the lines of VA improvement with BCVA. Three or more lines of improvement from UCVA to BCVA were found in 626 (30.9%) participants.

**Table 3:** Prevalence of uncorrected visual acuity (UCVA) with lines of visual acuity improvement in the better-seeing eye with best correction.

UCVA	Lines of Improvement with BCVA							Total
	0	1	2	3	4	5	≥6	
	n (%)	n (%)	n (%)	n (%)	n (%)	n (%)	n (%)	n (%)
20/20	406 (66.0)	0 (0.0)	0 (0.0)	0 (0.0)	0 (0.0)	0 (0.0)	0 (0.0)	406 (20.0)
20/25	48 (7.8)	288 (62.2)	0 (0.0)	0 (0.0)	0 (0.0)	0 (0.0)	0 (0.0)	336 (16.6)
20/32	22 (3.6)	64 (13.8)	182 (56.7)	0 (0.0)	0 (0.0)	0 (0.0)	0 (0.0)	268 (13.2)
20/40	20 (3.2)	34 (7.3)	53 (16.5)	123 (54.9)	0 (0.0)	0 (0.0)	0 (0.0)	230 (11.4)
20/50	19 (3.1)	24 (5.2)	29 (9.0)	41 (18.3)	76 (53.5)	0 (0.0)	0 (0.0)	189 (9.3)
20/63	10 (1.6)	19 (4.1)	15 (4.7)	23 (10.3)	27 (19.0)	64 (60.4)	0 (0.0)	158 (7.8)
20/80	13 (2.1)	12 (2.6)	19 (5.9)	12 (5.4)	13 (9.1)	10 (9.4)	42 (27.3)	121 (6.0)
20/100	6 (1.0)	9 (1.9)	9 (2.8)	6 (2.7)	11 (7.7)	12 (11.3)	36 (23.4)	89 (4.4)
20/125	3 (0.5)	9 (1.9)	5 (1.6)	11 (4.9)	6 (4.2)	7 (6.6)	31 (20.1)	72 (3.6)
20/160	4 (0.6)	1 (0.2)	3 (0.9)	1 (0.4)	5 (3.5)	2 (1.9)	15 (9.7)	31 (1.5)
20/200	1 (0.2)	2 (0.4)	2 (0.6)	4 (1.8)	2 (1.4)	8 (7.5)	16 (10.4)	35 (1.7)
20/250	0 (0.0)	0 (0.0)	2 (0.6)	1 (0.4)	1 (0.7)	1 (0.9)	4 (2.6)	9 (0.4)
20/320	2 (0.3)	0 (0.0)	2 (0.6)	0 (0.0)	0 (0.0)	0 (0.0)	5 (3.2)	9 (0.4)
20/400	7 (1.1)	1 (0.2)	0 (0.0)	1 (0.4)	1 (0.7)	1 (0.9)	3 (1.9)	14 (0.7)
<20/400	54 (8.8)	0 (0.0)	0 (0.0)	1 (0.4)	0 (0.0)	1 (0.9)	2 (1.3)	58 (2.9)
ALL	615 (30.4)	463 (22.9)	321 (15.9)	224 (11.0)	142 (7.0)	106 (5.2)	154 (7.6)	2025 (100.0)

UCVA – uncorrected visual acuity; BCVA – best-corrected visual acuity.

The lines of improvement with BCVA compared to UCVA ranged from 0 to 11 lines (mean = 1.94 ± 2.06); and ranged from 0 to 9 lines (mean = 1.36 ± 1.91) with BCVA compared to PVA. Improvement of three or more lines in UCVA with BCVA was found in 626 (30.9%) participants, and in 399 (19.7%) participants.

In 615 (30.4%) participants there was no change in VA in the better-seeing eye with BCVA, including 406 (20.0%) participants with 20/20 UCVA, 48 (2.4%) participants with 20/25 vision, 22 (1.1%) participants with 20/32 vision along with another 139 (6.9%) participants having visual impairment ≤20/40 (For those cases with visual impairment who could not benefit from refractive correction, the principal cause was cataract in 61.9%, followed by 7.9% glaucoma, other 6.5% retinal causes and 6.5% pterygium).

**Figure 3:**
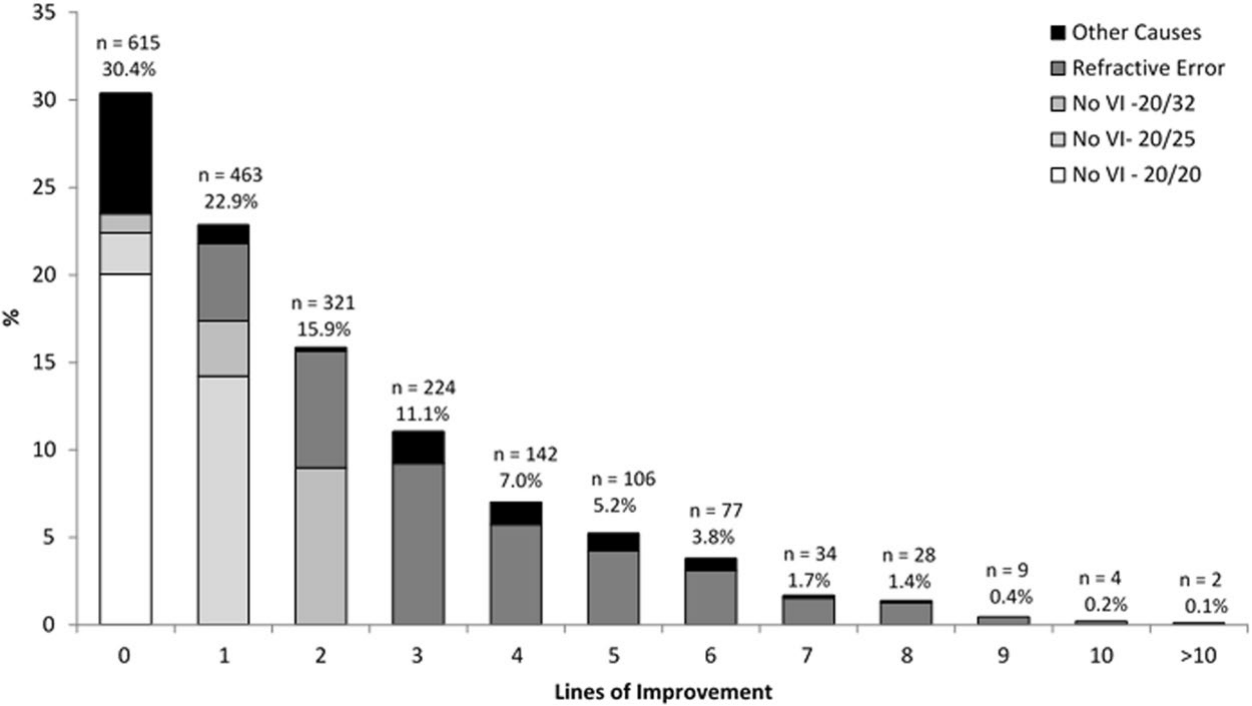
Change in lines from uncorrected visual acuity (UCVA) to best-corrected visual acuity (BCVA) in the better-seeing eye. Stacked bars show the distribution of eyes with lack of visual impairment (20/20; 20/25 and 20/32 visual acuities); eyes with refractive error only as the principal cause of visual impairment (includes only eyes improving to ≥20/32 with subjective refraction) and eyes with other causes as principal cause of visual impairment.

The change in lines in the better-seeing eye from UCVA to BCVA is shown in Fig. 3. In eyes with vision impairment (VA ≤ 20/40 in the better-seeing eye) improving to ≥20/32 vision with optical correction, refractive error was considered the main cause of vision impairment. Eyes with BCVA ≤ 20/40 in the better-seeing eye were considered as visually impaired due to mainly other causes even though refractive error could be present.

The association of VA improvement of three or more lines with BCVA in the better-seeing eye was investigated with multiple logistic regression with gender, age, schooling, and residence as co-variates. Female gender (OR, 1.44; 95% CI: 1.23–1.68) and rural residency (OR, 1.35; 95% CI: 1.06–1.78) were significantly associated with three or more lines of improvement from UCVA to BCVA in the better-seeing eye.

## Discussion

The strengths of this study were the large, randomly selected sample of participants and the fact that refraction was performed in all participants along with measurement of uncorrected, presenting (with glasses if they were used) and best-corrected (after subjective refraction) visual acuity from each eye under controlled conditions. To improve reliability and precision of visual acuity scores, clear instructions were given during the enumeration process for the participants to bring their own glasses to the examination station. The limitations to perform this survey were: (a) the distance and costly access to the city of Parintins; (b) the fact that the whole survey had to be executed during only four visits to maximize the permanence of the study team, the transport of equipment and availability of infrastructure for the ophthalmic exam and (c) the need to use a large boat to accommodate study team and equipment for 18 consecutive days to achieve the goal of testing residents in rural areas.

After adjusting for differences in age, gender, education and residency area, females, those with age between 65–74 years, those with higher education and those living in rural areas were more prone to come to the eye examination. These results are in line with previous studies in developing countries where female gender, older age and higher education were associated with participation in the eye exam^9^. Possible explanations for these findings take into account that women are more concerned about their healthcare than men, that older subjects have increased need for eye care services, that those with higher instruction have awareness of the importance of health care and that those in rural areas are in need due to lack of access. An additional observation related to the lack of access was that approximately only one third of the participants were wearing glasses, whereas in a similar study performed in Sao Paulo city 10 years earlier 51% participants were wearing glasses with distance correction^7^. Once again female gender and higher education were associated to any glasses usage. The fact that in Parintins city there was only one ophthalmologist residing in the city during the study period could explain partially these results. Consequently, refractive services were limited and available only to those who could afford to pay for a pair of glasses. To note, the urban areas of Parintins are not directly comparable to urban areas of a previous population-based study performed by our group in the city of São Paulo, the largest city in South America. In that sense, a more realistic classification for urban Parintins would be to consider it as semi-rural instead of urban.

The importance of including uncorrected visual acuity measurement in the study protocol is that uncorrected vision reflects a biological score of vision status, with presenting vision reflecting the services provided (since it was measured with glasses in 25% of participants) and best-corrected vision showing the optimal vision status to be reached with standard ophthalmic care. Vision status considering lines of visual impairment decreased approximately 50% both from uncorrected and presenting to best-corrected distance visual acuity. The decrease in vision impairment can also be confirmed by the results comparing uncorrected, presenting and best-corrected vision except to the severe blindness category in which similar results among the three measurements were found. These findings reinforce the strong impact of refractive services in this population with limited access to eye care providers. It is also remarkable that only 10% of the participants could reach 20/20 vision in both eyes with uncorrected visual acuity, with improvement to almost half of the sample considering the best-corrected vision. Sometimes, the worse-seeing eye with UCVA and with PVA may become the better-seeing eye after refraction, so that it is important to refract both eyes^10^. In 45 (2.2%) participants the better-seeing eye switched from UCVA to PVA, in 89 (4.4%) from PVA to BCVA and in 109 (5.4%) participants from UCVA to BCVA. These changes can be explained by differences in the magnitude of refractive error between eyes, inadequate optical correction in only one eye or presence of eye diseases considered optically uncorrectable as incipient cataract.

The availability of data from two ppulation-based studies performed in the last decade into two distinct socio-demographic areas: a low-income area of São Paulo city (SPES), the most industrialized city in the country along with urban and rural areas of Parintins city (BARES), in the central location of the Brazilian Amazon Region, allows comparisons that reflect regional and socio-economic disparities in the country. Even though SPES was performed 10 years earlier and in a population 5-years older than BARES population, the distribution of vision status categories between these two Brazilian studies using the same protocol are remarkable. Presenting normal vision (≥20/32) in the better-seeing eye was found in 78.1% in SPES and in 60.6% in BARES, increasing to 91.5% with best optical correction in SPES and to 81.4% in BARES. On the other hand, the prevalence of presenting severe blindness (<20/400) in the better-seeing eye was 0.77% in SPES and 2.81% in BARES, decreasing to 0.55% with best correction in SPES and to 2.67% in BARES. The fact that 2.67% remain blind despite the best optical correction is probably related to a substantial number of persons with irreversible blindness or with unoperated severe cataract. The inequalities of access to eye care services are substantial with people living in remote areas showing a five-fold prevalence of severe blindness than those living in low-income areas of developed cities.

Other population-based studies have showed optically correctable visual impairment in older adults using distinct criteria^11–13^. In the Australian study the change from visual impairment with PVA (<20/40 in the better-vision eye) to no visual impairment with BCVA (≥20/40 in the better vision eye) was found in 7.5% participants 49 years of age and older^11^. In Mexican-Americans 40 years and older, 6.0% had changed from visual impairment to no visual impairment with refractive correction^12^. In the Taiwanese study, 9.6% of participants (65 years of age and older) changed from visual impairment with PVA to no visual impairment with BCVA^13^. Applying this criterium to our data, 19.7% participants had optically correctable visual impairment. This finding might be related to the restricted access to refractive services in that remote part of Brazil.

The analysis of change in lines from presenting to best-corrected vision had been used in population-based studies^12^ and in the clinic to evaluate a large sample of patients with low vision^10^. In Mexican-Americans from Proyecto VER, out of 390 (8.2%) participants with visual impairment (PVA < 20/40 in the better-vision eye) a change in 2 or more lines from presenting to best-corrected vision was found in 299 (77%)^12^. In the current study, out of 604 (29.8%) participants with visual impairment (PVA < 20/40 in the better-vision eye), a change of 2 or more lines from PVA to BCVA was found in 398 (66%).

The current results demonstrate that around a third of the older adults from Parintins, Brazilian Amazon Region, obtained at least three lines of improvement in their visual acuity with proper refraction and approximately 20% of participants had optically correctable visual impairment. Sociodemographic parameters as female gender and rural residency were associated with three or more lines of visual acuity improvement by refraction. Based on these findings, it is important to reinforce to health authorities the need of sustainable actions to improve access to ophthalmic care along with provision of affordable glasses. The importance of regular eye examination and the possibility of improving visual acuity by wearing glasses should be improved to this population.

## References

[1] Resnikoff S, Pascolini D, Mariotti SP, Pokharel GP (2008). Global magnitude of visual impairment caused by uncorrected refractive errors in 2004. Bull. WHO..

[2] Stevens GA (2013). Ophthalmology..

[3] Klein R, Klein BEK, Linton KLP, DeMets DL (1991). The Beaver Dam Eye Study: visual acuity. Ophthalmology..

[4] Consultation on development of standards for characterization of vision loss and visual functioning. World Health Organization. http://apps.who.int/iris/bitstream/10665/68601/1/WHO_PBL_03.91.pdf (2003).

[5] ICD-10 - International statistical classification of diseases and related health problems. World Health Organization. http://apps.who.int/classifications/icd10/browse/2016/en#/H54.0. (2016).

[6] Salomão SR (2017). The Brazilian Amazon Region Eye Survey: design and methods. Ophthalmic Epidemiol..

[7] Salomão SR (2008). Prevalence and causes of vision impairment and blindness in older adults in Brazil: the São Paulo Eye Study. Ophthalmic Epidemiol..

[8] Stata Corporation. STATA/SE Statistical Software. Release 14.0. College Station, TX. Stata Corporation (2015).

[9] Zhao J (2010). Prevalence of vision impairment in older adults in rural China: the China Nine-Province Survey. Ophthalmology..

[10] Sunness JS, El Annan J (2010). Improvement of visual acuity by refraction in a low-vision population. Ophthalmology..

[11] Foran S, Rose K, Wang JJ, Mitchell P (2002). Correctable visual impairment in an older population: the Blue Mountains Eye Study. Am. J. Ophthalmol..

[12] Muñoz B (2002). Blindness, visual impairment and the problem of uncorrected refractive error in a Mexican-American population: Proyecto VER. Invest. Ophthalmol. Vis. Sci..

[13] Kuang T (2007). Correctable visual impairment in elderly Chinese population in Taiwan: The Shihpai Eye Study. Invest. Ophthalmol. Vis. Sci..

